# Beyond “Epidemiological Alchemy” in the Era of Open Data and Generative AI: Prepared Minds Still Matter

**DOI:** 10.2188/jea.JE20260079

**Published:** 2026-07-05

**Authors:** Kota Katanoda, Atsushi Goto

**Affiliations:** 1 Division of Population Data Science, National Cancer Center Institute for Cancer Control, Tokyo, Japan; 2 Department of Public Health, School of Medicine, Yokohama City University, Yokohama, Japan

In this issue, Ide et al published a letter entitled “Epidemiological Alchemy in the AI era comes under close scrutiny—but is it entirely without meaning?” drawing attention to an emerging concern in contemporary epidemiological research.^1^ The letter highlights a phenomenon in which technically correct but conceptually weak studies are rapidly produced by combining open datasets with generative artificial intelligence (AI). While the data and tools themselves are legitimate, their uncritical use can undermine the scientific value of epidemiological research.

Open science has long been promoted for its ability to enhance transparency, reproducibility, and equity in research.^2^ Large-scale datasets, such as the United States National Health and Nutrition Examination Survey (NHANES)^3^ and the Global Burden of Disease (GBD)^4^ study, have enabled researchers worldwide to explore important population health questions without the barriers of primary data collection. However, the rapid development of generative AI has introduced new challenges to this ecosystem.

## OPEN DATA MEETS GENERATIVE AI: A NEW CHALLENGE

The combination of open data and AI now allows the technical production of manuscripts at unprecedented speed, raising concerns about an increase in low-quality, redundant, or poorly conceptualized studies. Importantly, not all large datasets are fully open; many large-scale study groups, such as the UK Biobank^5^ and the J-MICC Study,^6^ allow access to their data resources on an application basis and impose governance structures that encourage thoughtful study design. In contrast, fully public datasets, while invaluable, are particularly vulnerable to superficial reuse.

As noted in the aforementioned letter,^1^ several journals have begun to respond by automatically rejecting certain categories of submissions or by requiring detailed checklists addressing study rationale and data use. For example, the *Journal of Global Health* asks authors to explain five specific aspects of their open-data-based analyses.^7^ Nevertheless, in the age of generative AI, even such explanations can be mechanically produced, raising doubts about their effectiveness as safeguards.

## OPPORTUNITIES FOR EARLY CAREER RESEARCHERS

At the same time, it is essential to recognize the positive potential of open data and generative AI. For early career researchers, fully publicly available datasets represent rare and valuable opportunities to gain research experience. Generative AI has also become a transformative tool for early-career researchers and non-native English speakers, substantially improving productivity and lowering barriers to participation in global science.^8^^,^^9^ Indeed, this editorial was drafted with the assistance of generative AI to enhance clarity and language.

Reflecting these considerations, the *Journal of Epidemiology* does not explicitly reject studies using open data, nor does it disallow the use of generative AI, provided that its use is appropriately disclosed.^10^ Despite this inclusive stance, a large number of manuscripts that used open data are submitted to the journal, many of which are desk-rejected.

## ADVICE FOR EARLY CAREER RESEARCHERS

Based on editorial experience, we offer three pieces of advice to early career researchers. First, researchers should recognize that research using open data is easy to start but difficult in practice. Generating originality from public datasets is challenging because such data are rarely collected to test specific hypotheses, and many research questions have already been extensively explored. The fact that data are accessible to everyone does not mean that the data are ready to produce publishable research.

Second, generative AI should be understood as a synthesis of past knowledge rather than a source of originality. Generative AI can assist with language, structure, and efficiency, but it cannot produce genuinely novel scientific ideas.

Third, collaboration with experienced researchers, particularly those who are deeply familiar with the characteristics and limitations of specific datasets, is crucial. All data have inherent structures, biases, and constraints, and without understanding these properties, no valid analysis and interpretation are possible. Generative AI often produces erroneous information unsupported by scientific evidence. The use of generative AI requires the presence of researchers capable of determining the authenticity of the generated content.

## A CALL TO EXPERIENCED RESEARCHERS AND EDUCATORS

Another piece of advice, or rather, a request, is directed toward experienced researchers and educators: let’s actively use open data and generative AI as educational tools. Open datasets provide excellent material for training in data cleaning, understanding data structures and characteristics, descriptive analysis, visualization, and feature extraction. Novice researchers are often unable to judge the validity of outputs generated by AI, and only experts who were trained before the AI era can provide reliable standards for such judgment. Educators can help beginners learn how to appraise AI outputs by asking questions such as: “Do you think the data are suitable for answering your research questions?”, “Are you sure that this statistical code produces the intended analytical results?”, “Can you explain the limitations or weaknesses of the AI outputs?” Data quality improves through use, scrutiny, and feedback. Collaboration across generations in the use of new technologies can create new value. Maintaining and utilizing open data is a scientific responsibility to society, encompassing not only data provision but also quality assurance and education. Academic institutions are well positioned to design systems that support this responsibility.

## RESEARCH WITHOUT GOOD QUESTIONS IS A WASTE

In the end, it comes down to Knottnerus and Tugwell’s statement: “A study without good questions is a waste.”^11^ Rather than starting with the question of what can be done with available open data, researchers should return to the first principle and focus on formulating meaningful questions. If the data required to answer those questions are difficult to access or collect, experienced researchers may help you open pathways to appropriate data or refine the question itself.

We conclude with the words of Louis Pasteur: “Chance favors the prepared mind.” In the era of open data and generative AI, chance should favor the prepared **human** mind—not merely prepared data or tools.
